# Cardiovascular Diseases: Exploring Epidemiology, Pathophysiology, Risk Factors, and Medicinal Plant‐Based Therapies: A Narrative Review

**DOI:** 10.1002/hsr2.73102

**Published:** 2026-08-26

**Authors:** Nawfal Hasan Siam, Umme Halima Mithila, Sadia Alam Tisha, Hridoy Saha, Ayesha Binte Mahbub, Farhan Nizam, Johirul Islam, Noushin Tabasumma, Md. Mazharul Islam Chowdhury

**Affiliations:** ^1^ Department of Pharmacy, School of Pharmacy and Public Health Independent University, Bangladesh (IUB) Dhaka Bangladesh; ^2^ Department of Pharmaceutical Chemistry, Faculty of Pharmacy University of Dhaka Dhaka Bangladesh; ^3^ Department of Pharmacy, School of Pharmacy University of Asia Pacific (UAP) Dhaka Bangladesh; ^4^ Department of Pharmaceutical Sciences Appalachian College of Pharmacy Oakwood Virginia USA

**Keywords:** atherosclerosis, cardiovascular disease, hyperlipidemia, hypertension, triacylglycerol

## Abstract

**Background and Aims:**

Cardiovascular diseases (CVDs) remain the leading cause of global morbidity and mortality, encompassing coronary artery disease, hypertension, heart failure, and cerebrovascular disorders. The global burden of CVD continues to rise, driven by complex interactions among endothelial dysfunction, oxidative stress, inflammation, genetic predisposition, and lifestyle‐related risk factors. This review aims to provide a comprehensive overview of the epidemiology, pathophysiology, risk factors, and emerging therapeutic approaches for CVD, highlighting recent advances in precision medicine and phytotherapy.

**Methods:**

A comprehensive literature search was conducted using PubMed, ScienceDirect, and Google Scholar databases. Keywords included “cardiovascular disease,” “Atherosclerosis,” “Pathophysiology,” “Risk Factors,” “Treatment,” “Hyperglycemia,” and “Hypertension.” Approximately 300 publications were initially identified. Following title, abstract, and full‐text screening, 156 relevant articles were selected for inclusion. About 85% of the reviewed literature was published between 2020 and 2025, while the remaining 15% originated from 2016 to 2019.

**Results:**

The review demonstrates a substantial increase in the global burden of CVDs, with prevalence nearly doubling between 1990 and 2019 and mortality continuing to rise worldwide. Atherosclerosis emerged as the primary pathological basis of ischemic cardiovascular disorders, driven by endothelial dysfunction, oxidative stress, inflammatory cytokines, and vascular remodeling. Major modifiable risk factors include hypertension, smoking, diabetes, dyslipidemia, obesity, unhealthy diet, alcohol consumption, and physical inactivity, while age, sex, and genetic susceptibility contribute to disease risk. Emerging therapeutic strategies such as PCSK9 inhibitors, dual SGLT1/2 inhibitors, siRNA‐based therapies, CRISPR/Cas9 genome editing, and anti‐inflammatory biologics show promising clinical outcomes. Additionally, medicinal plants including *Astragalus membranaceus*, *Citrus bergamia*, *Hibiscus sabdariffa*, and *Olea europaea* exhibit cardioprotective effects.

**Conclusion:**

CVD remains a significant global health challenge requiring multifaceted management strategies. The integration of conventional pharmacotherapy, precision medicine, gene‐based interventions, and evidence‐based phytotherapeutics offers promising opportunities to improve cardiovascular outcomes and reduce the growing global disease burden.

**Abbreviations**
ACE: angiotensin‐converting enzyme
AM: adrenomedullin
BP: blood pressure
COX‐1: cyclooxygenase‐1
CVD: cardiovascular diseases
DL: deep learning
DM: diabetes mellitus
HDL‐C: high‐density lipoprotein cholesterol
HTN: hypertension
ILs: intensive lifestyle interventions
LDL: low‐density lipoprotein
LDL‐C: low‐density lipoprotein cholesterol
miRs: microRNAs
MMPs: matrix metalloproteinases
MSCs: mesenchymal stem cells
PCSK9: proprotein convertase subtilisin/kexin type 9
ROS: reactive oxygen species
siRNA: small interfering RNA
SMCs: smooth muscle cells
TC: total cholesterol
TG: triglyceride
VTE: venous thromboembolism

## 1 Introduction

Cardiovascular diseases (CVDs) encompass a broad spectrum of disorders affecting the heart and blood vessels, including coronary artery disease (e.g., heart attack), hypertension (HTN), heart failure, cerebrovascular diseases (e.g., stroke), and other heart conditions [1]. Atherosclerosis, a coronary artery disease (CAD), represents the most prevalent type of CVD, which is characterized by the buildup of lipids and inflammation in the large arteries, resulting in clinical complications such as myocardial infarction (MI) and stroke [2].

CVDs are the leading noncommunicable conditions globally, responsible for about one‐third of all deaths worldwide [3]. The Global Burden of Disease, Injury, and Risk Factors Study (GBD) 2019 reported a dramatic increase in both the number of CVD cases (from 272 million to 523 million) and CVD‐related deaths (from 12.1 million to 18.6 million) between 1990 and 2019 [4]. By disrupting the balance of nitric oxide (NO), oxidative stress triggers chronic blood vessel wall inflammation and endothelial dysfunction, which drives the development and progression of atherosclerotic CVD [5]. Atherosclerosis, the underlying pathology of CVD, is significantly influenced by lipids and lipoprotein particles, which also impact inflammatory processes and the function of leukocytes, vascular, and cardiac cells, thereby affecting vessels and the heart [6]. The study found common risk factors for CVD, including a sedentary lifestyle, tobacco smoking, obesity, hyperlipidemia, diabetes mellitus (DM), HTN, and insufficient physical activity [7]. Dyslipidemia is a recognized contributor to the risk of CVDs [8].

First‐line treatment for HTN consists of lifestyle modifications (e.g., weight loss, exercise, low sodium). A wide range of synthetic agents, including anticoagulants, calcium channel blockers (CCBs), vasodilators, angiotensin‐converting enzyme (ACE) inhibitors, antiplatelet drugs, statins, β‐blockers, and protein‐based therapies, are employed in cardiovascular care but often impose significant adverse effects and high costs, driving a rapid rise in the use of herbal medicines. The World Health Organization (WHO) estimates that phytomedicines account for nearly 75% of the global medical market. Research on medicinal plants has therefore gained prominence, not only for their structural diversity and therapeutic potential but also for advancing the study of metabolites and their mechanisms of action. Beyond pharmacological effects, herbal remedies confer added value through nutritional support, owing to their abundance of bioactive phytochemicals and essential minerals [9]. Although numerous reviews have addressed individual aspects of CVD, most focus either on epidemiology and risk factors, molecular pathophysiology, conventional pharmacotherapy, or complementary medicine in isolation. The novelty of the present review lies in its integrative framework, which connects global epidemiological trends and atherosclerotic mechanisms with emerging precision‐cardiology interventions including Proprotein convertase subtilisin/kexin type 9 (PCSK9) inhibitors, small interfering RNA (siRNA) therapeutics, CRISPR/Cas9‐based strategies, and anti‐inflammatory biologics while critically examining evidence‐based phytotherapeutic approaches. By synthesizing these traditionally separate fields within a single clinically oriented review, this article provides a contemporary overview of how conventional pharmacology, molecular medicine, and plant‐derived therapeutics may collectively contribute to future cardiovascular prevention and management. The aim of this study is to summarize the epidemiology, pathophysiology, risk factors, and treatments of CVD.

## 2 Methodology

### 2.1 Literature Search Strategy

A comprehensive literature search was conducted using PubMed, ScienceDirect, and Google Scholar databases. The search employed combinations of the following keywords: “cardiovascular disease,” “atherosclerosis,” “pathophysiology,” “risk factors,” “hypertension,” “hyperglycemia,” “treatment,” “precision medicine,” “gene therapy,” and “phytotherapy.” A preliminary review of approximately 300 papers was conducted, after which around 156 articles were shortlisted through an initial screening for detailed evaluation and inclusion in this review.

**Figure 1:**
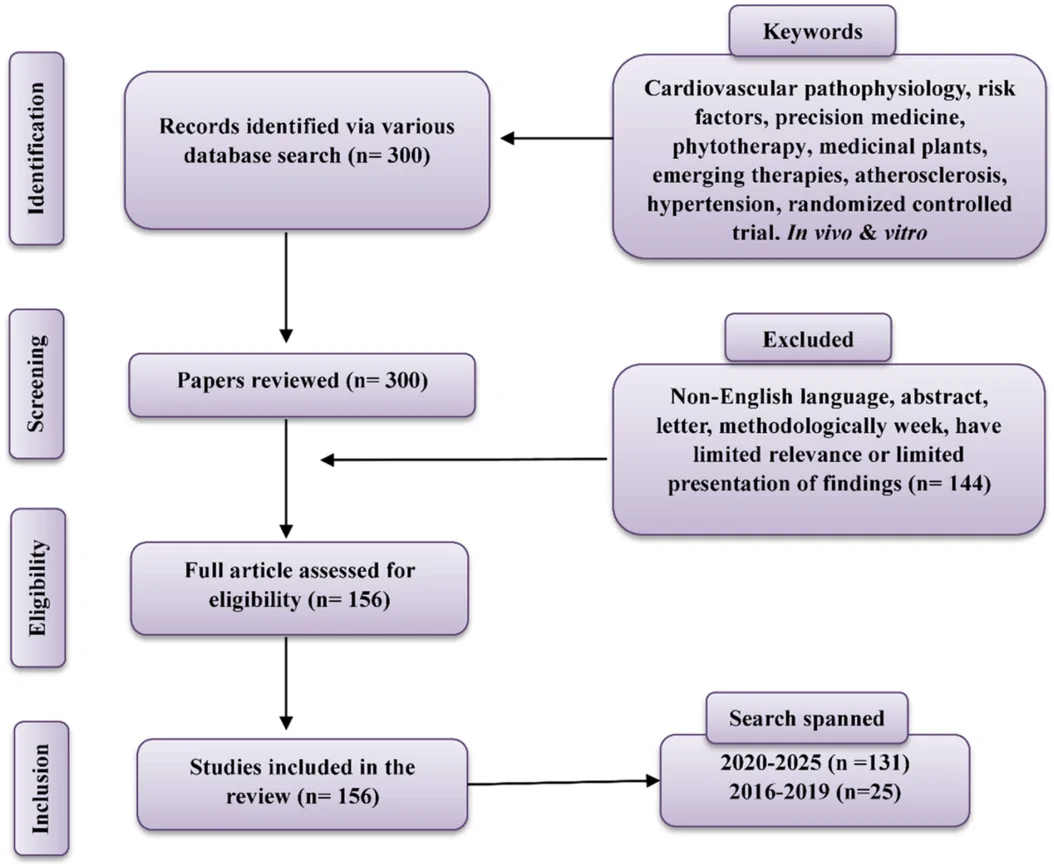
Flow diagram illustrating the review process for studies on cardiovascular disease. This figure illustrates the four stages of the systematic review process: identification, screening, eligibility, and inclusion and exclusion. The diagram also provides an overview of the search periods.

Priority was given to studies published between 2020 and 2025 (approximately 85%) to ensure inclusion of the most recent advances in cardiovascular research, particularly in precision medicine, RNA‐based therapeutics, gene‐editing technologies, and emerging phytotherapeutic strategies. Earlier studies published between 2016 and 2019 (approximately 15%) were included when they represented landmark investigations, foundational mechanistic evidence, major clinical guidelines, or pivotal epidemiological reports essential for contextualizing recent findings (Figure 1).

Studies were included if they focused on CVDs, including two, hypertension, heart failure, stroke, and atherosclerosis, and examined aspects such as epidemiology, pathophysiology, risk factors, conventional treatments, emerging therapeutic strategies, and medicinal plant‐based interventions. Eligible studies included original research articles, clinical trials, systematic reviews, meta‐analyses, and high‐quality observational studies published in peer‐reviewed journals and available in the English language.

Studies were excluded if they were conference abstracts, editorials, letters, dissertations, or duplicate publications. Articles lacking sufficient methodological details, outcome data, or scientific rigor were also excluded. Additionally, studies focusing on non‐cardiovascular conditions without direct relevance to CVD management and publications written in languages other than English were not considered for inclusion in this review.

## 3 Epidemiology

Congestive MI, heart failure, stroke, and ischemic heart disease are just a few of the many conditions that fall under the umbrella of CVD. From 1990 to 2019, the number of CVD cases doubled from 271 million to 523 million [10]. An estimated 207 million Indians suffer from HTN, according to estimates from the fourth District‐Level Household Survey, which was conducted between 2012 and 2014 [11]. Among 53 studies involving 305,432 subjects, the overall weighted pooled prevalence of hypertension was 20.0% as of 2019 in Bangladesh [12]. In 2018, there were an estimated 51.6 million Koreans and 126.8 million Japanese, of whom an estimated 12 million and 43 million, respectively, suffered from HTN [13]. The China Hypertension Survey (CHS), conducted between 2012 and 2015, found that 23.2% of Chinese adults aged ≥ 18 had HTN, with a weighted prevalence of 27.9%, and estimated that 245 million adults had HTN in China [14]. The overall age‐adjusted weighted prevalence of HTN in Pakistan was 46.2%. In both urban and rural regions, the weight prevalence of HTN was 44.3% and 46.8%, respectively [15]. 49.64% (95% confidence interval [CI]: 46.67–52.61) of people had hypertension in 2017–2018, representing 115 (95% CI: 104–128) million people in the USA [16]. Using the American Heart Association (AHA) standards, the National Health and Nutrition Survey (Ensanut) 2020 found that 49.4% (44.0% of women and 55.3% of men) of Mexican adults had HTN [17]. The South Africa Demographic and Health Survey 2016 reported that the prevalence of hypertension, as per the Joint National Committee 7 guidelines, was 50.4% (95% CI: 48.3%, 52.5%), while it rose to 75.0% (95% CI: 73.1%, 76.7%) according to the 2017 American College of Cardiology/American Heart Association (ACC/AHA 2017) guidelines [18]. Similarly, the CUORE Project cross‐sectional health survey conducted in Italy in 2018–2019 found the prevalence of hypertension to be 44% in men and 32% in women among individuals aged 35–74 years [19]. According to a meta‐analysis conducted by the Turkish Society of Cardiology, the overall prevalence of HTN was revealed to be 31.2% [20]. According to the European Health Interview Survey (3, 2018–2020), the estimated prevalence in Germany was higher compared to the European average: CVDs 6.8% versus 5.7% [21]. In Spain, 33% (10 million) of adults aged 30–79 suffered from HTN in 2018 [22]. There were 119.3 cases of age‐standardized hypertensive heart disease (HHD) per 100,000 individuals (95% uncertainty interval 86.6–161.0) in 1990; in 2019, that number was 80.1 cases (95% UI 57.4–108.1) in Australia [23]. According to the GBD Study 2019, 6.1% of Brazilians were projected to have CVD [24]. Collectively, these findings highlight the substantial global burden of CVD and its major risk factors, particularly hypertension, while also demonstrating considerable regional variation in prevalence across different populations [10, 11, 12, 13, 14, 15, 16, 17, 18, 19, 20, 21, 22, 23, 24].

## 4 Pathophysiology

**Figure 2:**
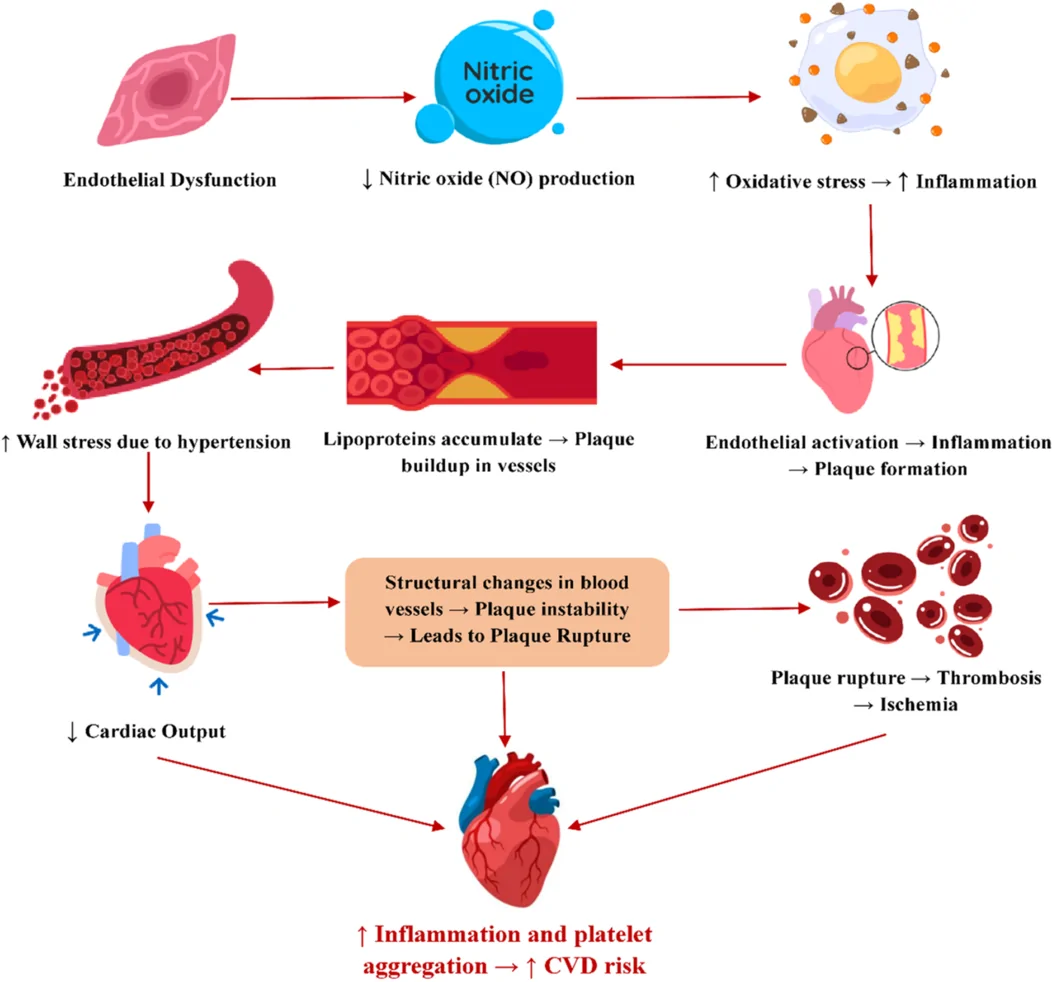
Pathophysiological events in the progression of cardiovascular diseases (CVD). This figure depicts the progression of CVD beginning with reduced nitric oxide (NO) production and increased oxidative stress, leading to endothelial dysfunction. Lipoprotein accumulation, inflammation, and hypertension promote plaque formation and vessel narrowing. Plaque instability and rupture can trigger thrombosis and ischemia, ultimately reduce cardiac output and increase cardiovascular risk.

CVDs encompass a range of disorders affecting the heart and vascular system, with atherosclerosis recognized as the primary underlying cause [25]. Endothelial dysfunction plays a critical role in these conditions, leading to impaired NO–mediated vasodilation, reduced cellular glucose uptake, heightened oxidative stress, and chronic inflammation [26]. The initiation of atherosclerosis begins with endothelial activation, triggering a cascade of events that result in vessel narrowing and the activation of inflammatory pathways, culminating in the formation of atheromatous plaques [27]. This inflammatory response in cardiovascular tissues is characterized by elevated levels of pro‐inflammatory cytokines such as IL‐1β and TNF‐α, secreted by resident and infiltrating immune cells, alongside a concurrent decrease in anti‐inflammatory cytokines like IL‐10 and TGF‐β [28]. These inflammatory and endothelial alterations not only initiate atherosclerotic plaque formation but also trigger a series of structural, cellular, and molecular changes within the cardiovascular system that drive disease progression and clinical manifestations. Cardiac hypertrophy, which develops during HTN, initially serves as a compensatory response to maintain cardiac output in the face of increased wall stress [29]. Vascular remodeling, a hallmark of CVD progression, involves structural alterations in the blood vessels, including changes in size, shape, and cellular and molecular composition [30]. Apoptosis and senescence of smooth muscle cells (SMCs), as well as their transformation into macrophage‐like cells, may exacerbate atherosclerosis by promoting inflammation. The extracellular matrix plays a pivotal role in atherosclerosis initiation through interactions between negatively charged proteoglycans and positively charged apolipoproteins, facilitating the retention of plasma‐derived lipoproteins [31]. The hallmark of atherosclerosis is the formation of plaques, with vascular occlusion caused by plaque rupture serving as a critical pathophysiological event in ischemic CVD [32]. The upregulation of adhesion molecules such as intercellular adhesion molecule‐1, vascular cell adhesion molecule‐1, and various selectins promotes the adhesion, rolling, and transmigration of inflammatory cells like monocytes and T helper cells at early plaque sites [33]. Expression of IL‐6 mRNA and protein has been observed within atherosclerotic plaques and arterial walls, highlighting the inflammatory milieu [34]. The biological activities of inflammatory mediators contribute to plaque development and rupture, endothelial dysfunction, and ultimately coronary thrombosis [35]. Obstructive sleep apnea, a common sleep disorder, influences multiple neural, metabolic, thrombotic, and inflammatory pathways implicated in CVD pathogenesis. Platelet aggregation, a marker of vascular dysfunction, is associated with increased CVD risk [36]. Under physiological conditions, low levels of reactive oxygen species (ROS) are balanced by detoxification systems, playing key roles in cellular signaling, especially in atherosclerosis. However, excessive ROS production surpasses antioxidant defenses, contributing to disease progression [37]. Vulnerable atherosclerotic plaques prone to rupture exhibit increased infiltration of inflammatory cells, leading to collagen degradation via collagenolytic enzymes, primarily matrix metalloproteinases (MMPs) and lipoprotein‐associated phospholipase A2, while also reducing collagen synthesis by promoting SMC apoptosis [38]. Lipid peroxidation products such as polyunsaturated fatty acid hydroperoxides may further exacerbate endothelial dysfunction by increasing ROS production, decreasing NO availability, and sustaining chronic inflammation in macrophages [39]. Consequently, understanding the complex pathophysiology of CVD is illustrated in Figure 2.

## 5 Risk Factor

CVD develops through the interaction of multiple modifiable and non‐modifiable risk factors. The American Society for Preventive Cardiology Top Ten CVD Risk Factors 2021 Update provides an annual evidence‐based summary of the most important factors contributing to CVD risk [40]. Major modifiable risk factors for CVD include cigarette smoking, high blood pressure (BP), lipid abnormalities, and DM, which are critical in disease onset and progression [41]. However, BP quantification has lacked sufficient granularity and precision to fully capture the CVD risk associated with hypertension [42]. Systolic BP levels below 115 or 120 mmHg are currently used to define normal SBP levels, yet it remains unclear whether there is a threshold below which the risk for atherosclerotic CVD plateaus or increases [43]. The risk for CVD escalates with both the concentration and duration of exposure to low‐density lipoprotein cholesterol (LDL‐C), which is universally regarded as a primary modifiable risk factor [44], and is included in most global cardiovascular risk prediction models [45]. T2DM remains a well‐established major risk factor for CVD, with inflammatory markers linked to the disease further exacerbating cardiovascular risk [46]. T2DM patients are approximately twice as likely to experience cardiovascular events related to atherosclerosis [47]. Similarly, obesity independently induces changes in cardiac structure and function, significantly raising the risk of CVD [48]. A sedentary lifestyle is another key modifiable risk factor, with individuals showing favorable behavioral responses benefiting the most from intensive lifestyle interventions (ILs), thus reducing long‐term CVD risk [49]. Notably, female smokers exhibit a 25% higher risk of developing CVD compared to their male counterparts, and CVD mortality in women is higher than in men [50]. The ASCVD Risk Estimator Plus suggests that former smokers should have the same CVD risk as never‐smokers after 5 years [51]. The combination of central obesity and smoking results in a 3.04‐fold increase in CVD risk, with smokers possessing central hypertension, high triglycerides, low high‐density lipoprotein cholesterol (HDL‐C), and obesity facing the highest risk, showing an odds ratio of 14.18 [52]. Poor diet and physical inactivity are well‐documented behavioral risk factors for CVD incidence and mortality [53]. Polygenic risk scores, which combine multiple single nucleotide polymorphisms, have proven effective in predicting CVD risk and can reflect the multifactorial nature of the disease [54]. Excessive alcohol consumption, defined as > 60 g/day in men and > 40 g/day in women, is a significant contributor to CVD mortality and burden, with up to 19% of alcohol‐attributable deaths linked to CVD [55, 56]. Mendelian randomization has confirmed a causal relationship between alcohol intake and increased CVD risk [57]. Atherosclerosis, a primary cause of CVD, is characterized by chronic inflammation of large‐to‐medium‐sized arteries [58]. The impact of chronic stress, whether physical, chemical, or psychological, on CVD development and acute cardiac events is well documented [59]. The primary contributor to CVD is atherosclerosis, characterized by chronic inflammation of the vessel wall of large‐to‐medium‐sized arteries [60]. CVD is preventable, and optimal treatment based on absolute risk can halve the risk of future cardiovascular events. However, men generally face a higher CVD risk compared to women, with CVDs accounting for approximately 52% of all deaths among women in Europe according to the WHO [61, 62]. Preventive medications are recommended for patients at high short‐term CVD risk, although many younger individuals with multiple risk factors may present low short‐term risk [63]. Age plays a critical role in cardiovascular deterioration, with the prevalence of CVD increasing in older adults, including atherosclerosis [64]. Women, particularly, face an excess CVD risk compared to men [65]. A family history of CVD increases the likelihood of future CVD in offspring, and age‐adjusted analyses have shown higher CVD incidence rates in individuals with a parental history of CVD, although this correlation was not statistically significant for all risk factors except smoking [66]. A family history of ASCVD serves as an accessible marker of genetic cardiovascular risk [67]. Genetic predispositions, such as those associated with smoking, also increase the risk of various forms of CVD [68]. While integrating genetic risk into clinical practice holds promise, the results regarding its impact on CVD management have been mixed [69].

## 6 Treatment

### 6.1 Current Therapy

Rheumatic stroke, heart disease, HHD, and ischemic heart disease are subtypes of CVD, which is the most common chronic disease and a leading global cause of death and disability [70]. The most widely used cardiovascular medications are antihypertensive drugs, diuretics, statins, nitrate esters, antiarrhythmics, anticoagulants, and antiplatelet medicines [71]. Beta‐blockers are used to treat angina pectoris, arrhythmias, and heart failure, whereas ACE inhibitors offer cardioprotection in acute coronary syndromes and congestive heart failure [72]. Cilazapril, Benazepril, enalapril, captopril, fosinopril, enalaprilat, perindopril, lisinopril, quinapril, trandolapril, moexipril, ramipril are listed as ACE inhibitors [73]. ACE inhibitors inhibit the enzyme responsible for converting angiotensin (AT) I to AT II, preventing the synthesis of AT II, resulting in arterial and venous vasodilation, natriuresis, decreased sympathetic activity, and a decrease in BP [74]. Cardioselective beta‐blockers that antagonize β1 receptors comprise metoprolol, bisoprolol, atenolol, and nebivolol, and non‐cardioselective beta‐blockers that antagonize β1 and β2 receptors comprise carvedilol, labetalol, pindolol, propranolol, nadolol, and timolol [75]. CCBs such as Clevidipine, amlodipine, benidipine, efonidipine, nimodipine, and paradipine act as antihypertensive agents by inhibiting the influx of calcium ions into vascular smooth muscle, resulting in dilation of arteries and a decrease in BP [76]. AT II receptor blockers, or ARBs, are renin‐angiotensin‐aldosterone system blocking drugs that are often used as first‐line treatments for a significant number of hypertensive patients [77]. Candesartan, fimasartan, irbesartan, losartan, olmesartan, telmisartan and valsartan are listed as ARBs; among them, losartan is the most commonly used drug [78]. Losartan is a widely recognized AT1 receptor blocker that inhibits AT1 receptors and intracellular calcium signaling, facilitating blood vessel dilation [79]. Inhibiting the sodium‐chloride co‐transporter in the distal convoluted tubule, hydrochlorothiazide, a common thiazide diuretic, lowers BP, promotes potassium loss and diuresis, reduces myocardial fibrosis, and helps prevent heart failure [80]. Nitroglycerin, a fast‐acting vasodilator that converts into NO and causes venodilation at low doses and arteriolar dilation at high doses, is used for the treatment of angina, acute coronary syndromes, heart failure, and aortic dissection [81]. Statins’ beneficial effects on lipid profiles, anti‐inflammatory properties, and other plaque‐stabilizing actions make them effective in preventing CVD‐related morbidity and mortality [82]. Statins such as simvastatin, atorvastatin, rosuvastatin, and pravastatin significantly reduced non‐fatal stroke, MI, and cardiovascular death by lowering non‐HDL cholesterol [83].

### 6.2 Emerging Therapies

Currently, targeted therapies, including protein drugs, gene editing technology, nucleic acid drugs, and cell therapy, are applied to treat CVD [84]. PCSK9 protein levels in the blood can be therapeutically inhibited with CRISPR/Cas9, which lowers the risk of cardiovascular illnesses, and CRISPR/Cas9 knockout of the angptl3 gene may lower the risk of CVD [85]. In regenerative medicine, stem cell‐based therapy has recently become a major option for treating several diseases, including the use of multipotent mesenchymal stem cells (MSCs) in CVD [86]. MSCs protect the myocardium by reducing inflammation, promoting myocardial cell differentiation around infarct areas and angiogenesis, increasing apoptosis resistance, and inhibiting fibrosis to repair the cardiovascular system [87]. In the case of ribonucleic acid (RNA)‐based therapeutics, inhibition of microRNAs (miRs) such as miR‐15 or miR‐34 may provide therapeutic benefits by increasing cardiomyocyte proliferation, regulating cardiac remodeling, and controlling the effects of aging on cardiovascular regeneration [88]. Antithrombotic drugs, such as antiplatelets and anticoagulants, are effective in the prevention and management of various cardiovascular conditions, including venous thromboembolism (VTE), acute coronary syndrome, and stroke [89]. Aspirin, a cyclooxygenase‐1 (COX‐1) inhibitor, and platelet adenosine diphosphate P2Y12 receptor inhibitors such as ticagrelor, prasugrel, and clopidogrel are examples of oral antiplatelet medicines [90]. Aspirin irreversibly inhibits COX‐1 through acetylation of serine 529, reducing the synthesis of prostaglandin G2/H2 and thromboxane A2 for platelet lifespan, blocking arachidonic acid access to the enzyme's active site, acetylating lysine residues on fibrinogen, increasing fibrin clot permeability and promoting clot lysis [91]. Anticoagulants, including warfarin and the direct oral anticoagulants such as rivaroxaban and apixaban, are used for a wide range of thrombotic disorders, to prevent stroke and systemic embolism associated with atrial fibrillation, and to prevent or treat VTE [92].

### 6.3 Novel Therapeutic Agents

Recent advances in cardiovascular therapeutics have shifted toward precision medicine, harnessing molecular and genetic approaches to address key drivers of atherosclerosis, heart failure, and related complications. Several novel agents are currently in advanced stages of development, while some have already entered clinical practice, showing promising efficacy in reducing major adverse cardiovascular events (Table 1).

**Table 1:** Novel therapeutic agents under development for cardiovascular disease (CVD) management.

Therapy	Approach	Target/benefit	Status	Ref
Lepodisiran	Small interfering RNA (siRNA)	↓ Mean serum concentrations of lipoprotein(a)	Phase 3 ongoing	[93]
Sotagliflozin (Inpefa)	SGLT1/SGLT2 inhibitor	↓ Myocardial infarction (MI), stroke, and heart failure (HF)	Approved	[94]
VERVE‐101 gene therapy	CRISPR base editing	↓ LDL cholesterol	Fast track; phase 1b ongoing	[95]
Nexiguran Ziclumeran (CRISPR for ATTR‐CM)	CRISPR gene editing	Transthyretin (TTR) reduction, improved symptoms	Phase 3 ongoing	[96]
Inclisiran	siRNA	↓ LDL, ↓ acute coronary syndrome, ↑ concentration of HDL‐C	Approved	[97]
Obicetrapib	CETP inhibitor	LDL↓, HDL ↑, potential Lp(a) ↓	Phase 3 trials ongoing	[97]
Pacibekitug	Anti‐IL‐6 monoclonal antibody	Reduces inflammation, high‐sensitivity C‐reactive protein (hs‐CRP)	Phase 2 trials ongoing	[98]

Lepodisiran, a siRNA, is designed to specifically lower serum concentrations of lipoprotein(a), an established independent risk factor for atherosclerotic CVD. A phase 3 clinical trial is ongoing to validate its long‐term efficacy and safety [93]. Similarly, inclisiran, another siRNA, has demonstrated potent LDL‐cholesterol lowering along with reduction in acute coronary syndromes and elevation in HDL‐C, leading to its regulatory approval in several countries [97]. Another major therapeutic advance is the dual SGLT1/SGLT2 inhibitor sotagliflozin (Inpefa), which has shown benefits in reducing MI, stroke, and heart failure hospitalization, expanding the clinical utility of SGLT inhibitors beyond glycemic control and earning FDA approval [94]. In contrast, obicetrapib, a next‐generation CETP inhibitor, is currently under phase 3 investigation, with data suggesting significant LDL reduction and HDL elevation, as well as a potential effect on lowering lipoprotein(a) [97]. Gene editing technologies are also being rapidly translated into cardiovascular therapeutics. VERVE‐101, a CRISPR base‐editing therapy, offers a one‐time intervention aimed at durable reduction of LDL cholesterol, and is currently undergoing phase 1b evaluation under FDA Fast Track designation [95]. Likewise, Nexiguran Ziclumeran, a CRISPR‐based therapy targeting transthyretin (TTR), has demonstrated promising results in transthyretin amyloid cardiomyopathy, with ongoing phase 3 trials reporting improvements in functional outcomes [96]. Beyond lipid‐centric therapies, modulation of inflammation is emerging as a critical strategy. Pacibekitug, an anti‐IL‐6 monoclonal antibody, is under phase 2 clinical evaluation, showing the potential to reduce vascular inflammation and systemic hs‐CRP levels, thereby mitigating residual inflammatory risk in CVD [98].

Despite their promising therapeutic potential, several challenges remain. Many of these emerging therapies are associated with high development and treatment costs, which may limit accessibility and widespread implementation, particularly in low‐ and middle‐income countries. Long‐term safety and durability data are still limited for novel modalities such as siRNA‐based therapies and CRISPR/Cas9 gene editing, necessitating extended clinical follow‐up. In addition, potential off‐target effects, immunogenicity, regulatory complexities, and ethical concerns surrounding gene‐editing technologies require careful evaluation. Furthermore, the clinical efficacy of some agents remains under investigation in ongoing phase 2 and phase 3 trials, highlighting the need for robust evidence before routine incorporation into standard cardiovascular care. Collectively, these investigational and recently approved therapies represent a paradigm shift in cardiovascular management, targeting genetic, inflammatory, and metabolic pathways that were previously untreatable. Their success would pave the way toward precision cardiology, with durable benefits in preventing MI, stroke, and heart failure.

### 6.4 Alternative Treatment

**Figure 3:**
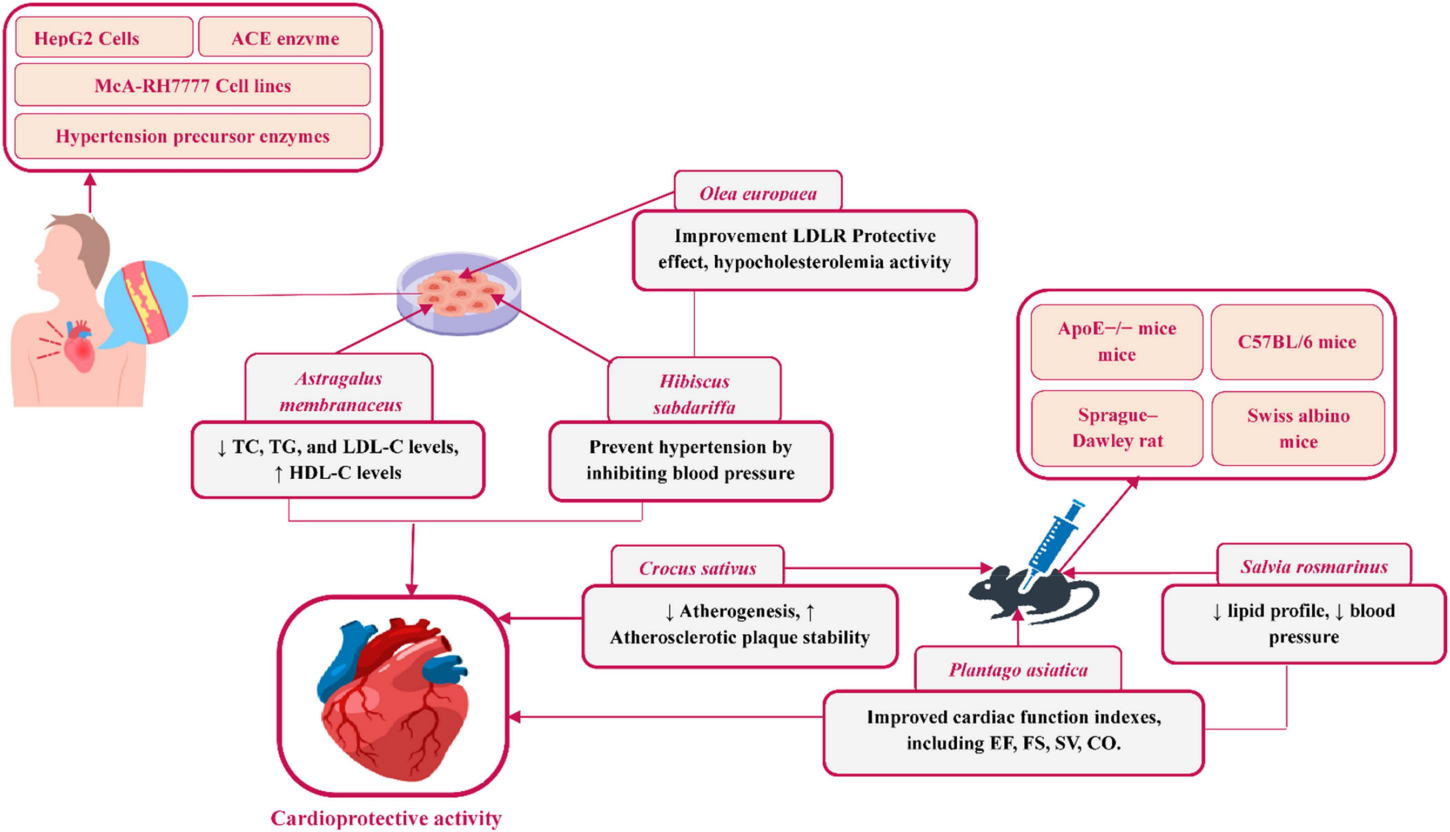
Proposed mechanisms of cardioprotective activity of medicinal plants. This figure summarizes the proposed in vitro and in vivo mechanisms through which medicinal plants exert cardioprotective effects that improve lipid metabolism by reducing total cholesterol, triglycerides, and LDL‐cholesterol while increasing HDL‐cholesterol. Additional mechanisms include inhibition of angiotensin‐converting enzyme (ACE), reduction of blood pressure, suppression of oxidative stress and reactive oxygen species (ROS) generation, attenuation of inflammatory signaling, improvement of endothelial function, enhancement of antioxidant defenses, stabilization of atherosclerotic plaques, and protection against cardiac hypertrophy and vascular injury.

Various medicinal plants have been traditionally used to address numerous diseases, and many exhibit potential benefits for cardiovascular conditions. Notably, plants like *Astragalus membranaceus* Moench, *Cydonia oblonga* Mill*., and Crocus sativus* L. have shown effectiveness in combating CVD by significantly lowering triacylglycerol (TG), total cholesterol (TC), LDL, and increasing HDL. Table 2 provides an overview of preclinical trials exploring the therapeutic potential of these and other medicinal plants in managing CVD and related metabolic disorders such as hypertension and dyslipidemia. These studies focused on specific plant‐based interventions and their outcomes, including improvements in lipid profiles, TC, BP regulation, and antioxidant activity. The results indicate promising pharmacological effects, suggesting that these plants could play a crucial role in the prevention and treatment of CVD and its associated conditions. Proposed in vivo and in vitro mechanisms of action of cardioprotective activity are given in Figure 3.

**Table 2:** Medicinal plant used in CVD.

Plant name	Parts	Preparation type/extract	Study type	Study models/methods	Dose administration	Effects/result	Ref
*Astragalus membranaceus* Moench		Methanol	In vitro	*HepG2 cells*	200 μM	↓ TC, TG, and LDL‐C levels, ↑ HDL‐C levels, ↓ liver injury, and ↑ antioxidant capacity	[99]
			In vivo	Induced high‐fat diet, *KM mice*	41.25, 82.5 and 165 mg/kg		
	Roots	Distilled water	In vivo	High‐fat diet containing 10% lard, 15% egg yolk powder, 1% cholesterol, and 76% basic diet, *Sprague–Dawley rats*	50, 100, and 150 mg/kg	↓ TC, TG, and LDL‐C levels, ↑ HDL‐C levels	[100]
*Cydonia oblonga* Mill.	Fruit and leaves	Ethanol	In vivo	Induced hyperlipidemia, Sprague–Dawley SPF rats	40,80,160 mg/kg	↓ Serum TC, TG, LDL‐C, ALT and AST, its potential value in the prevention and treatment of hyperlipidemia	[101]
	Fruit	Methanolic	In Vivo	Doxorubicin‐induced cardiotoxicity, Wistar rats	160 and 320 mg/kg	↑ Lipid profile and cardioprotective activity	[102]
	Leaves	Ethanol	In vivo		0.1 mg/mL	Vasorelaxation and ↓ vasoreactivity	[103]
*Crocus sativus* L.		Aqueous	In vivo	Induced high‐fat diet, *ApoE* ^*−/−*^ mice	30, 60, 90 mg/kg	↓ Atherogenesis, ↑ atherosclerotic plaque stability and improves glucose	[104]
			In vivo	Induced hypercholesterolemia, C57BL/6 mice		↓ Cholesterol, triglycerides, and ROS, reversed fatty liver degeneration, and downregulated PCSK9 and sortilin expression while upregulating LDLR	[105]
		Methanol		Rat cardiomyoblast cell line H9c2	0.1–100 µM	Potential application in the prevention of cardiovascular diseases	[106]
	Flower	Ethanol	In vivo	Induced established atherosclerosis, rabbits	50 and 100 mg/kg	Therapeutic potential in atherosclerosis, ↓ vascular inflammation and endothelial activation may contribute to improved lipid profiles, ↓ atherosclerotic lesion severity, and ↑Plaque stability	[107]
*Hibiscus sabdariffa* L.		Aqueous	In vivo	Induced hypertension, Sprague–Dawley rats	30 mg	↓ Blood pressure (BP)	[108]
		Distilled water	In vitro	Angiotensin‐converting enzyme (ACE) and hypertension precursor enzymes		Prevent hypertension by inhibiting blood pressure‐regulating enzymes such as ACE	[109]
	Flower	Methanol	In vivo	Guinea‐pigs	0.01–10 mg/mL	High cardiac and vascular protective activities	[110]
	Flower	Aqueous	In vivo	Induction of hypertension, Wistar rats	250 mg/kg	↓ High BP, antihypertensive effect ↓ plasma ACE, angiotensin II, and aldosterone levels	[111]
*Olea europaea* L.	leaves	Ethanol	In vivo	Induced hypercholesterolemia, Swiss albino mice	200 mg/kg	↓ The risk of atherosclerosis. ↓ TC, LDL, VLDL, and TG and ↑ HDL	[112]
	leaves	Ethanol	In vivo	Induced high‐fat diet, rabbits	150 mg/kg	↓ Total cholesterol, low‐density lipoprotein, total triglycerides	[113]
	—	—	In Vitro	HepG2 cells	25 μg/mL	↓ LDLR receptor (LDLR) protective effect on cardiovascular system, hypocholesterolemia activity	[114]
	Leaves	Methanol	In vivo	Guinea‐pigs	0.01–10 mg/mL	High cardiac and vascular protective activity	[110]
*Plantago asiatica* L.	Seeds	Ethanol	In vivo	Spontaneously hypertensive rats (SHR)	400 mg/kg	Potential antihypertensive effect	[115]
			In vitro		200 μg/mL		
	Seeds	Seed extract	In vivo	Induced high‐fat diet, C57BL/6 mice	1.44 g/kg	↓ Abdominal white adipose tissue ratio, white/brown adipocyte size, serum total cholesterol, TG, low‐density lipoprotein cholesterol, free fatty acid, and hepatic TG concentrations	[116]
	Seed	Seed extract	In vivo	Spontaneously hypertensive Wistar rats	0.36 g/kg	↓ BP	[117]
		—	In vivo	Induced cardiac hypertrophy, C57BL/6 mice	20, 40, and 80 mg/kg	↑ Cardiac function indices, including ejection fraction (EF), fractional shortening (FS), stroke volume (SV), and cardiac output (CO)	[118]
*Salvia rosmarinus* Spenn.	Aerial parts	Ethanol	In vitro	McA‐RH7777 (rat Morri's hepatoma‐derived cell line)	25, 50, 100, and 150 μg/mL	Antioxidant activity, anti‐lipidemic activity, ↓ lipid accumulation	[119]
	Leaves		In vivo	Induced hyperlipidemia, mice	0.11 gm	↓ Lipid profile, ↓ TC, TG and LDL, ↑HDL‐C	[120]
*Syzygium polyanthum* (Wight) Walp.	Leaves	Ethanol	In vivo	—	400, 600, and 800 mg/kg	↓ Cholesterol, triglyceride, and LDL levels and ↑ HDL	[121]
		Methanol	In vivo	Normotensive WKY and SHR rats	2, 2.50 and 3 g/kg	Antihypertensive effects	[122]
*Vitis vinifera* L.		Aqueous	In vivo	Spontaneously hypertensive Wistar Rats	200 mg/kg	↓ BP, antioxidant effect, improvement in lipid	[123]
	Fruits	Methanol	In vivo	Spontaneously hypertensive Rats	150 and 300 mg/kg/day	↓ BP levels, ↑ vascular resistance	[124]
	leaves	Methanol	In vivo	Induction of hypercholesterolemia, Wistar rats	100, 200 and 400 mg/kg	↓ Cholesterol level, ↑ HDL, ↓ disruption of endothelial lining and thickness of blood vessel lining, anti‐atherosclerosis and anti‐dyslipidemia activities	[125]
*Zingiber officinale* Roscoe	Rhizomes	Aqueous and methanolic	In vivo	Induced high‐fat diet, mice	250 and 500 mg/Kg.	↓ TC, LDL, VLDL, TG, and ↑ HDL treat hyperlipidemia and cardiovascular complications.	[126]
		Petroleum ether	In vivo	Hypertensive rats (SHRs)	250 mg/kg	Vascular relaxation mechanisms involve nitric oxide and prostacyclin release, activation of cGMP‐KATP channels, ↑ muscarinic receptors, and ↓ calcium influx. Potential antihypertensive properties	[127]
		—	In vivo	Induced hypertension, Wistar rats	75 mg/Kg	↑ Antihypertensive effect	[128]

*A. membranaceus* Moench (family Fabaceae) has been extensively studied for its lipid‐lowering and hepatoprotective potential. In an in vitro study using HepG2 cells, methanolic extracts of *A. membranaceus* at a concentration of 200 μM significantly reduced TC, TG, and LDL‐C levels, while concomitantly increasing HDL‐C. In addition, the extract mitigated liver injury and enhanced antioxidant capacity, suggesting a multifaceted protective effect against dyslipidemia and oxidative stress [99]. Consistent with these findings, in vivo experiments using Kunming (KM) mice fed a high‐fat diet demonstrated that oral administration of *A. membranaceus* at doses of 41.25, 82.5, and 165 mg/kg effectively improved lipid metabolism and reduced hepatic lipid accumulation [99]. Furthermore, studies on the root extract prepared in distilled water confirmed its beneficial activity in Sprague–Dawley rats maintained on a high‐fat diet containing 10% lard, 15% egg yolk powder, and 1% cholesterol. Treatment at doses of 50, 100, and 150 mg/kg markedly decreased serum TC, TG, and LDL‐C levels, while increasing HDL‐C, further underscoring its therapeutic promise in hyperlipidemia [100].

Similarly, *C. oblonga* Mill. (family Rosaceae), commonly known as quince, has demonstrated significant cardioprotective and hypolipidemic activities. Ethanol extracts of its fruits and leaves, when administered to hyperlipidemic Sprague–Dawley SPF rats at doses of 40, 80, and 160 mg/kg, reduced serum TC, TG, LDL‐C, as well as hepatic injury markers such as alanine aminotransferase and aspartate aminotransferase, highlighting its potential for both lipid regulation and hepatoprotection [101]. Complementary evidence comes from methanolic fruit extracts evaluated in Wistar rats with doxorubicin‐induced cardiotoxicity, where treatment at 160 and 320 mg/kg ameliorated lipid dysregulation and provided cardioprotective benefits [102]. In addition, ethanol extracts of *C. oblonga* leaves at 0.1 mg/mL produced pronounced vasorelaxation and reduced vasoreactivity, suggesting that its vascular effects may also contribute to cardioprotection [103].

*C. sativus* L. (family Iridaceae), widely known as saffron, has shown considerable potential in ameliorating cardiovascular risk factors. Aqueous extracts administered to ApoE−/− mice on a high‐fat diet at doses of 30, 60, and 90 mg/kg reduced atherogenesis, enhanced plaque stability, and improved glucose homeostasis [104]. Parallel in vivo experiments in hypercholesterolemic C57BL/6 mice demonstrated that saffron treatment lowered cholesterol, TG, and ROS, reversed fatty liver degeneration, and modulated cholesterol metabolism by downregulating PCSK9 and sortilin expression while upregulating LDLR [105]. Mechanistic in vitro studies using H9c2 rat cardiomyoblast cells confirmed that methanolic extracts at 0.1–100 μM could protect against cardiomyocyte injury, highlighting saffron's relevance in CVD prevention [106]. Moreover, in rabbit models of established atherosclerosis, ethanolic flower extracts (50 and 100 mg/kg) significantly attenuated vascular inflammation, decreased endothelial activation, improved lipid profiles, reduced lesion severity, and enhanced plaque stability, reinforcing saffron's therapeutic potential in atherosclerosis management [107].

*H. sabdariffa* L. (family Malvaceae), widely recognized for its therapeutic potential in cardiovascular health, has been extensively studied in both in vivo and in vitro models. In Sprague–Dawley rats with induced hypertension, administration of aqueous extracts at a dose of 30 mg resulted in a significant reduction of BP, suggesting its direct antihypertensive potential [108]. Further in vitro studies have elucidated the underlying mechanism, demonstrating that the aqueous extract effectively inhibits ACE and other hypertension precursor enzymes, thereby preventing the enzymatic regulation that elevates BP [109]. Methanolic extracts of *H. sabdariffa* flowers in guinea pigs at concentrations ranging from 0.01 to 10 mg/mL exhibited strong cardioprotective and vasculoprotective effects, reinforcing its vascular health benefits [110]. Moreover, aqueous flower extracts administered at 250 mg/kg in Wistar rats with experimentally induced hypertension markedly lowered BP by reducing plasma ACE, angiotensin II, and aldosterone levels, confirming its antihypertensive mechanism through modulation of the renin–angiotensin–aldosterone system [111].

*Olea europaea* L. (family Oleaceae), commonly known as the olive tree, is another plant with remarkable cardiovascular protective effects. Ethanol extracts of *O. europaea* leaves (200 mg/kg) administered to Swiss albino mice with hypercholesterolemia significantly reduced TC, LDL, very‐low‐density lipoprotein (VLDL), and TG, while simultaneously elevating HDL levels, thereby reducing atherosclerotic risk [112]. Similarly, in rabbits fed a high‐fat diet, leaf ethanol extract at 150 mg/kg demonstrated significant hypolipidemic activity by reducing TC, LDL, and TG concentrations [113]. In vitro investigations using HepG2 cells revealed that *O. europaea* extracts (25 μg/mL) upregulated LDL receptor (LDLR) expression, indicating a protective role against hypercholesterolemia through enhanced cholesterol clearance [114]. Methanolic leaf extracts also exhibited profound cardioprotective and vasculoprotective effects in guinea pigs, further supporting its role in vascular health [110].

*Plantago asiatica* L. (family Plantaginaceae), another traditional medicinal herb, has been studied for its antihypertensive and cardiometabolic regulatory activities. Ethanol extracts of *P. asiatica* seeds at 400 mg/kg in spontaneously hypertensive rats (SHR) significantly lowered BP, potentially through ACE inhibition, while concurrently preventing hypertension‐mediated organ damage [115]. In vivo studies using C57BL/6 mice subjected to a high‐fat diet revealed that seed extract at 1.44 g/kg markedly decreased abdominal white adipose tissue ratio, adipocyte size, and serum lipid parameters, including TC, TG, LDL‐C, and hepatic TG levels, demonstrating its lipid‐lowering efficacy [116]. In addition, oral administration of seeds at 0.36 g/kg in spontaneously hypertensive Wistar rats significantly lowered BP [117]. Importantly, in C57BL/6 mice with induced cardiac hypertrophy, seed extracts at 20–80 mg/kg improved cardiac performance, as evidenced by enhanced ejection fraction, fractional shortening, stroke volume, and cardiac output, thereby providing strong evidence for its cardioprotective role [118].

*Salvia rosmarinus* Spenn. (family: Lamiaceae), commonly known as rosemary, has demonstrated both in vitro and in vivo efficacy against dyslipidemia and oxidative stress. Ethanolic extracts from its aerial parts significantly reduced lipid accumulation in McA‐RH7777 rat hepatoma‐derived cells in a concentration‐dependent manner (25, 50, 100, and 150 μg/mL), suggesting potent antioxidant and anti‐lipidemic activity [119]. In animal models, oral administration of 0.11 g of *S. rosmarinus* leaves in hyperlipidemic mice resulted in a marked decrease in serum TC, TG, and LDL‐C, while simultaneously elevating HDL‐C, thereby confirming its lipid‐modulating potential [120].

Similarly, *Syzygium polyanthum* (Wight) Walp. (family: Myrtaceae) has emerged as another promising candidate for cardiovascular protection. In vivo administration of its ethanolic extract at doses of 400, 600, and 800 mg/kg produced significant reductions in cholesterol, triglycerides, and LDL‐C, with a concomitant increase in HDL‐C levels, highlighting its hypolipidemic potential [121]. Moreover, methanolic extracts of *S. polyanthum* leaves, tested in both normotensive Wistar Kyoto and SHR, revealed dose‐dependent (2.00, 2.50, and 3.00 g/kg) antihypertensive effects, thereby suggesting dual benefits in lipid regulation and BP control [122].

The cardioprotective properties of *Vitis vinifera* L. (family: Vitaceae), commonly known as grape, have also been widely validated in experimental models. An aqueous extract of *V. vinifera* administered at 200 mg/kg protected against the development of hypertension in spontaneously hypertensive Wistar rats, an effect partly attributed to enhanced superoxide dismutase activity and subsequent antioxidant defense. Furthermore, improvements in the lipid profile were reported, which likely contributed to its protective cardiovascular effects [123]. In another study, methanolic extracts of *V. vinifera* fruits at doses of 150 and 300 mg/kg/day produced a significant reduction in BP in both hypertensive and normotensive rats, along with improved vascular resistance [124]. Additionally, methanolic extracts of grape leaves at doses of 100, 200, and 400 mg/kg effectively reduced serum cholesterol and increased HDL‐C levels in hypercholesterolemic Wistar rats. These extracts also demonstrated anti‐atherosclerotic and anti‐dyslipidemic properties by reducing endothelial disruption and vascular wall thickening [125].

Among spices, *Zingiber officinale* Roscoe (family: Zingiberaceae), commonly known as ginger, has shown notable therapeutic potential in the prevention of cardiovascular complications. Both aqueous and methanolic extracts of ginger administered at doses of 250 and 500 mg/kg in mice fed a high‐fat diet reduced serum TC, LDL‐C, VLDL‐C, and TG, while increasing HDL‐C levels, confirming its role in the management of diet‐induced hyperlipidemia [126]. Furthermore, the petroleum ether extract of ginger rhizomes (250 mg/kg) exerted vasorelaxant effects through multiple mechanisms, including NO and prostacyclin release, activation of cGMP–KATP channels, muscarinic receptor stimulation, and reduced calcium influx, indicating significant antihypertensive potential [127]. Consistent with these findings, administration of 75 mg/kg of ginger extract in hypertensive Wistar rats enhanced antihypertensive activity, reinforcing its therapeutic role in vascular health and BP regulation [128].

### 6.5 Clinical Trial

A growing body of evidence from recent randomized, double‐blind, placebo‐controlled clinical trials underscores the multifaceted cardioprotective effects of medicinal plants. These studies highlight several mechanisms of benefit, including BP regulation, lipid modulation, antioxidant defense, vascular protection, and anti‐inflammatory activity, all of which contribute to reducing overall cardiovascular risk (Table 3).

**Table 3:** Summary of clinical trials evaluating the cardioprotective effects of medicinal plants.

Plant's scientific names	Study model	Administered material	Duration	Subjects	Health condition of subjects	Dose	Result	Ref
*Allium sativum* L. (Black Garlic, optimized ABG10+ extract)	Triple‐blind, randomized, placebo‐controlled clinical trial	Tablet	12 weeks	67	Grade I hypertension under drug treatment	250 mg daily	↓ Systolic (−1.8 mmHg) and diastolic (−1.5 mmHg) BP ↑ antioxidant, ↓uric acid and ACE activity	[129]
*Beta vulgaris* L.	A placebo‐controlled, double blind, randomized controlled trial	Beetroot juice	60 days	47	—	—	↓ Blood pressure and attenuating CVD risk	[130]
*Berberis* L.	A randomized, double‐blind, placebo‐controlled crossover design.	Berberine (capsule)	30 days	19	Healthy participants	1000 mg daily	↓ TC	[131]
*Astragalus membranaceus* Moench	Double‐blind, randomized crossover design	—	12 weeks	40	Hypertensive patients with dyslipidemia	16 mg daily	↓ Blood pressure, ↑ plasma HDL cholesterol	[132]
*Camellia sinensis* (L.) Kuntze	A randomized double‐blinded placebo‐controlled trial	Green tea beverage	6 weeks	60	Patients with dyslipidemia	—	↓ Total cholesterol, LDL, lipid peroxidation marker	[133]
*Citrus bergamia* Risso	A randomized, double‐blind placebo‐controlled clinical trial	Capsule	4 months	64	Patients with high cholesterol	375 mg daily	↓ TC and LDL‐C, ↑ HDL‐C	[134]
*Coffea arabica L*.	Randomized, double‐blind, placebo‐controlled trial	Capsules	10 weeks	44	Patients with T2D and overweight/obesity	400 mg twice per day	↓ Systolic blood pressure (SBP), TG, ↑ high‐density lipoprotein (HDL)	[135]
*Cynara cardunculus* L. + *Citrus bergamia* Risso	A parallel‐design, randomized, double‐blind, placebo‐controlled clinical trial	Tablets	12 weeks	90	Healthy individuals with suboptimal cholesterol levels	Artichoke (700 mg) and Bergamot (375 mg)	Significant improvement in TC, LDL‐C, Non‐HDL‐C, TG, Apo B‐100, Apo AI, glucose, alanine transaminase (ALT), gamma‐glutamyl transferase (gGT), hs‐CRP	[136]
*Curcuma longa* L.	A parallel‐design, randomized, double‐blind, placebo‐controlled clinical trial	Nano‐curcumin capsule	12 weeks	42	Overweight or obese patients with coronary slow flow phenomenon	80 mg/day	Improve disease‐related physical and mental complications, including angina stability, ↓ TC and LDL‐C, ↑HDL	[137]
*Hibiscus sabdariffa* L.	Double‐blind, randomized, placebo‐controlled clinical trial	Aqueous calyx extract	12 weeks	108	Abdominal obesity and mild metabolic syndrome (MetS)	1000 mg/day capsule	↓ LDL	[1, 138]
*Terminalia arjuna* (Roxb. ex‐DC.) Wight & Arn.	A randomized, double‐blind, placebo‐controlled, parallel‐group trial	TA bark extract (capsules)	12 weeks	120	Patients with stage 1–2 hypertension	500 mg twice daily	↓ BP and improved antioxidant	[139]
*Punica granatum* L.	Double‐blind, randomized, placebo‐controlled parallel trial	Capsules	12 weeks	86	—	370 mg twice a day	↓Inflammatory markers and blood pressure	[140]
*Rosmarinus officinalis* L	A pilot, non‐randomized clinical trial	Tea bags (preparing the infusion)	45‐day	90	Individuals with grade 1 hypertension	2 g daily	↓ Systolic blood pressure (SBP), diastolic blood pressure (DBP)	[141]
*Vitis labruscana* Bailey	A randomized, double‐blind, placebo‐controlled trial	Leaf extract (HP‐01 tablet)	12‐week	80	Participants with CVD risk factors	1800 mg/day	↓ Platelet aggregation by closure time, ↑ activated partial thromboplastin time (aPTT), ↓ SBP	[142]

#### 6.5.1 BP Regulation

Multiple trials demonstrated that plant‐derived interventions exert modest but clinically meaningful antihypertensive effects. *Allium sativum* (black garlic) supplementation reduced both systolic and diastolic BP in patients with grade I hypertension already receiving treatment, while also decreasing uric acid and ACE activity, suggesting dual vascular and renal protective actions [129]. Similarly, *Beta vulgaris* (beetroot) juice lowered BP through enhanced NO bioavailability [130], while *Terminalia arjuna* bark extract significantly improved BP and antioxidant status in hypertensive patients [139]. *Rosmarinus officinalis* tea, though evaluated in a smaller pilot design, yielded consistent reductions in systolic and diastolic pressures, expanding the evidence base for dietary herbs in hypertension management [141]. Collectively, these findings indicate that certain plant extracts may offer adjunctive benefits alongside pharmacotherapy, particularly in early‐stage or borderline hypertensive patients.

Several interventions targeted lipid profiles, which remain central to cardiovascular risk reduction. *Citrus bergamia* (bergamot) demonstrated strong lipid‐lowering effects, significantly reducing TC and LDL‐C while increasing HDL‐C [134]. Similarly, *Camellia sinensis* (green tea) reduced TC, LDL, and lipid peroxidation markers, consistent with its polyphenol‐driven hypolipidemic and antioxidant effects [133]. *Berberis* spp. (berberine) reduced TC even in healthy individuals [131], highlighting its potential preventive role. *A. membranaceus* was particularly notable for its dual action of lowering BP and improving HDL levels in hypertensive patients with dyslipidemia [132]. The combined formulation of *Cynara cardunculus* (artichoke) and bergamot produced the broadest cardiometabolic benefits, improving lipid fractions, apolipoproteins, glucose, liver enzymes, and inflammatory markers [136]. This suggests synergism between plant‐derived bioactives when used together, though it complicates attribution to a single compound.

Beyond conventional lipid and BP endpoints, some trials investigated vascular reactivity and hemostatic balance. *Vitis labruscana* (grape‐leaf extract) supplementation reduced platelet aggregation and prolonged activated partial thromboplastin time (aPTT) while lowering systolic BP [142], pointing to antithrombotic effects with implications for atherosclerotic and ischemic disease prevention. This mechanistic pathway, shared with polyphenol‐rich plants like grapes and pomegranates, supports vascular protection beyond traditional risk factor modulation. Inflammatory and oxidative stress pathways. Chronic inflammation and oxidative imbalance are central to cardiovascular pathology, and several plants in these trials showed meaningful activity in these domains. Black garlic improved antioxidant capacity [129], nano‐curcumin from *Curcuma longa* improved disease‐related symptoms and favorably modulated lipid parameters in coronary slow flow patients [137], and *Punica granatum* (pomegranate) supplementation lowered inflammatory markers and BP [140]. These findings reinforce the relevance of anti‐inflammatory phytochemicals in conditions where conventional therapies have limited impact, such as microvascular dysfunction and low‐grade systemic inflammation. Clinical implications and translational relevance. Although the effect sizes observed in these studies were modest compared to pharmaceutical interventions, the consistency across diverse populations hypertensive, dyslipidemic, obese, and metabolically impaired patients suggests that plant‐derived therapies hold substantial potential as complementary strategies. Importantly, most interventions were well tolerated, supporting their safety in long‐term use. The diversity of mechanisms ranging from modulation of lipid metabolism, NO pathways, antioxidant defenses, to platelet inhibition underscores the potential of plant‐based compounds to address multiple cardiovascular risk factors simultaneously, a feature rarely achievable with single pharmacological agents (Table 3).

#### 6.5.2 Clinical Implications and Translational Relevance

Despite these promising findings, several challenges limit the translation of medicinal plants into routine cardiovascular practice. Considerable variability exists in plant species, cultivation conditions, extraction procedures, and phytochemical composition, which can affect reproducibility and therapeutic efficacy. In addition, dosages used across preclinical and clinical studies vary substantially, making direct comparisons and evidence‐based dosing recommendations difficult (Tables 2, 3). Although many plant‐derived interventions have demonstrated favorable safety profiles, most clinical studies are limited by relatively small sample sizes, short follow‐up periods, and heterogeneous study designs. Furthermore, potential herb–drug interactions and the lack of standardized regulatory frameworks warrant careful consideration. Therefore, large‐scale, well‐designed clinical trials using standardized formulations are required before these phytotherapeutic agents can be widely integrated into evidence‐based cardiovascular care (Table 3).

## 7 Future Research and Direction

Mitochondrial dysfunction is increasingly acknowledged as a fundamental factor in the development of CVDs, including heart failure, ischemic heart disease, HTN, and cardiomyopathy [143]. This dysfunction critically affects cardiovascular health by disrupting ATP production, which is essential for cellular energy. A key pathway through which mitochondrial impairment contributes to CVD is the elevation of oxidative stress [144]. Excessive mitochondrial ROS generation is a major pathological mechanism in CVDs. Damage to mitochondrial DNA (mtDNA) can induce mutations that compromise the electron transport chain (ETC), thereby amplifying ROS production and establishing a detrimental feedback loop of mitochondrial dysfunction and oxidative damage [145]. Timely and precise diagnosis of CVD is vital for effective treatment and better patient outcomes. Emerging biomarkers such as microRNAs, myeloperoxidase, and growth differentiation factor‐15 (GDF‐15) show promise in improving early detection and risk stratification [146]. In addition, MMPs and their inhibitors have been proposed as valuable predictors for future cardiovascular events [147]. Advanced imaging modalities like cardiac magnetic resonance imaging offer detailed visualization of cardiac and vascular structures, facilitating the identification of anatomical abnormalities and functional impairments linked to CVDs. Computed tomography angiography also holds potential for early diagnosis [148]. AI is increasingly applied to cardiovascular healthcare challenges, including automated identification of cardiac arrhythmias from electrocardiograms, early recognition of aortic stenosis, and mortality risk prediction in patients receiving cardiac resynchronization therapy [149]. Although AI has initiated a digital transformation in CVD prediction, it remains in an early developmental stage, hindered by limitations in research design and evaluation frameworks [150]. AI methodologies such as machine learning, deep learning (DL), and cognitive computing are poised to play pivotal roles in early CVD detection, diagnostic accuracy, outcome prediction, and prognosis assessment [151]. Particularly, AI‐driven analysis of data from wearable sensors is crucial for enhancing diagnosis and prognostication in cardiovascular medicine [152]. Metabolic disturbances in obesity, including increased cardiac lipid accumulation, contribute significantly to cardiovascular complications. The adipose‐derived cytokine adiponectin plays a key role in metabolic disorders that elevate cardiac mortality risk [153]. Lysosomal dysfunction has been implicated not only in rare genetic disorders such as lysosomal storage diseases but also in common age‐related conditions, including cardiovascular and neurodegenerative diseases. Lysosomal membrane permeabilization, a regulated partial disruption of lysosomal membranes, modulates NLRP3 inflammasome activation under specific conditions [154]. Given its vasodilatory properties and consistent expression in the cardiovascular system, adrenomedullin was initially considered a promising therapeutic candidate for CVDs [155]. The identification of novel drug targets and development of next‐generation therapeutics hold promise for patients with CVDs who show poor response or intolerance to current regimens. Systematic investigation of herb–drug synergies may enhance efficacy and mitigate adverse effects, but requires validation through rigorous clinical trials. Long‐term studies are also essential to clarify sustained cardiovascular benefits, safety profiles, and the influence of diet, lifestyle, and environmental factors on therapeutic outcomes [156].

## 8 Conclusion

CVDs remain the leading cause of mortality and disability worldwide, with their increasing burden driven by hypertension, diabetes, obesity, dyslipidemia, smoking, and sedentary lifestyles. The complex pathophysiology of CVD involves atherosclerosis, endothelial dysfunction, chronic inflammation, oxidative stress, and maladaptive vascular remodeling, which collectively contribute to MI, stroke, and heart failure. Although conventional pharmacological therapies, including ACE inhibitors, β‐blockers, statins, and anticoagulants, have substantially improved cardiovascular outcomes, limitations related to residual cardiovascular risk, adverse effects, and treatment costs underscore the need for more effective therapeutic strategies. Recent advances in precision medicine, including siRNA‐based therapeutics, CRISPR/Cas9 gene editing, monoclonal antibodies targeting inflammatory pathways, and regenerative approaches, are reshaping the future of cardiovascular care by enabling more personalized and mechanism‐based interventions. In parallel, phytotherapeutic agents such as *A. membranaceus*, *C. bergamia*, *H. sabdariffa*, and *O. europaea* have demonstrated promising lipid‐lowering, antihypertensive, antioxidant, and anti‐atherosclerotic activities, suggesting their potential as complementary therapies alongside conventional treatment. Future research should focus on conducting large‐scale, multicenter randomized clinical trials to establish the long‐term efficacy, safety, and cost‐effectiveness of emerging molecular therapies and evidence‐based phytotherapeutics. In addition, integrating genomic profiling, novel biomarkers, artificial intelligence, and advanced cardiovascular imaging into clinical practice may improve early diagnosis, individualized risk prediction, and therapeutic decision‐making. Further investigation of herb–drug interactions, combination therapies, and implementation strategies across diverse populations will also be essential for translating these advances into routine cardiovascular care. Collectively, the integration of precision medicine, innovative pharmacological agents, validated phytotherapeutics, and personalized preventive strategies has the potential to transform CVD management, improve patient outcomes, and reduce the growing global cardiovascular burden.

## Author Contributions

**Nawfal Hasan Siam:** conceptualization, investigation, writing – original draft, writing – review and editing, visualization, validation, formal analysis, project administration, supervision, data curation, resources, funding acquisition. **Umme Halima Mithila:** writing – original draft, writing – review and editing, data curation. **Sadia Alam Tisha:** writing – original draft, writing – review and editing, data curation. **Hridoy Saha:** writing – original draft, writing – review and editing, data curation. **Ayesha Binte Mahbub:** writing – original draft, data curation, writing – review and editing. **Farhan Nizam:** writing – original draft, writing – review and editing, data curation. **Johirul Islam:** data curation, funding acquisition, writing – review and editing, writing – original draft. **Noushin Tabasumma:** writing – original draft, writing – review and editing, data curation, supervision, project administration, formal analysis. **Md. Mazharul Islam Chowdhury:** writing – review and editing, data curation, resources, project administration, supervision.

## Funding

The authors have nothing to report.

## Ethics Statement

The authors have nothing to report.

## Conflicts of Interest

The authors declare no conflicts of interest.

## Transparency Statement

The lead author, Nawfal Hasan Siam, affirms that this manuscript is an honest, accurate, and transparent account of the study being reported; that no important aspects of the study have been omitted; and that any discrepancies from the study as planned (and, if relevant, registered) have been explained.

## Data Availability

The authors have nothing to report.
